# Evaluation of the Safety and Efficacy of the Respiratory Syncytial Virus FG Chimeric Vaccine KD-409 in Rodent Models for Maternal and Pediatric Vaccination

**DOI:** 10.3390/vaccines13111170

**Published:** 2025-11-18

**Authors:** Ryo Yamaue, Madoka Terashima, Kenji Soejima, Masaharu Torikai

**Affiliations:** Kikuchi Research Center, KM Biologics Co., Ltd., 1314-1 Kyokushi Kawabe, Kikuchi-shi 869-1298, Japan; yamaue-ryo@kmbiologics.com (R.Y.); terashima-ma@kmbiologics.com (M.T.); soejima@kmbiologics.com (K.S.)

**Keywords:** respiratory syncytial virus, RSV, pediatric vaccine, maternal vaccine, safety, efficacy, FG chimeric protein, KD-409

## Abstract

Background/Objectives: Respiratory syncytial virus (RSV) causes severe infection in neonates and infants. However, a suitable RSV vaccine for children is yet to be approved. The development of KD-409 is focused on creating an effective and safe RSV vaccine for newborns and children. The safety and efficacy of the RSV FG chimeric protein KD-409 were evaluated in several rodent models. Methods/Results: The effect of vaccine-induced antibody transfer was verified in a guinea pig model. Next, the exacerbation of infection was evaluated in a BALB/c mouse model of passive immunity designed to mimic the vaccination of pregnant women. KD-409 did not exacerbate infection when administered with alum, unlike pre-F with alum. Our active immunization model of BALB/c mice, which involved stimulating vaccination with a pediatric vaccine, suggested that KD-409 with alum was less likely to exacerbate inflammation than FI-RSV or pre-F with alum. The efficacy was evaluated in a cotton rat model, in which KD-409 demonstrated greater protection against infection than pre-F without adjuvant, the only currently approved formulation for immunizing pregnant women. Conclusions: KD-409 eliminated concerns about vaccine-enhanced disease in pediatric vaccination and demonstrated superior efficacy to current vaccines in rodent models. The safety in mice during passive and active immunization, and efficacy in cotton rats demonstrate the high potential of KD-409 as a safe and effective next-generation RSV vaccine candidate that can cover the neonatal-to-pediatric age range.

## 1. Introduction

Respiratory syncytial virus (RSV) infection is more severe in neonates and infants. In 2019, an estimated 3.6 million RSV-acute lower respiratory infection (ALRI) hospitalizations and 101,400 RSV-ALRI deaths occurred in children aged 0–60 months worldwide [1], with an estimated 1.4 million RSV-ALRI hospitalizations and 45,700 RSV-ALRI-attributable deaths reported in infants aged 0–6 months [1]. RSV vaccines for children were developed in the 1960s using FI-RSV as an antigen; however, the vaccinated group exhibited adverse events such as vaccine-enhanced disease (VED) [2]. Despite the challenges associated with RSV vaccine development for half a century, the strategy of preventing infection with transferable antibodies after vaccination of pregnant women has been successful. The Food and Drug Administration (FDA) approved the first RSV vaccine for neonates on 21 August 2023 [3]. Nevertheless, the lower respiratory tract infections requiring medical care reduced by 57% (for 90 days) and 51% (for 6 months), showing no significant difference compared with the placebo group [4,5]. Further improvement in efficacy has been anticipated. Additionally, RSV vaccine development for children is ongoing, with clinical trials focusing on live attenuated vaccine candidates. However, suitable candidates are yet to be selected.

In the development of RSV vaccines, safety assurance is essential due to concerns regarding VED. Three causes of symptom exacerbation in natural infection after FI-RSV vaccination have been reported: (i) low avidity of induced antibodies [6]; (ii) Th2 induction by carbonylated proteins resulting from formalin treatment [7]; and (iii) Th2 induction by G proteins [8]. Issues i and ii are deemed avoidable with the appropriate use of adjuvants, recombinant proteins, or attenuated live viruses. In the case of (iii), using only the F protein as an antigen may be a suitable solution, although VED caused by adjuvanted F has been documented in some animal experiments [9]. Given that anti-G protein antibodies possess neutralizing activity [10], G proteins, as immune antigens, appear to be promising in terms of efficacy. The CX3C motif, which functions as both a host cell adhesive and an aggravating factor, is located at residues G182–186 within the conserved central domain (CCD; G157–198), surrounded by cysteine loops (G173–186) and a highly conserved region [11,12]. A clinical trial of BBG2Na, which fused the RSV A G130–230 residues (G2Na) containing this CCD region to the albumin-binding domain of streptococcal G protein (BB), was terminated following the occurrence of unexpected side effects [13].

Although the pre-F protein antigen vaccine is promising, several preclinical studies have indicated increased inflammation following RSV exposure after immunization [9]. Understanding the inflammatory response is crucial for developing RSV vaccines. Severe cases of neonatal RSV infection are common up to 6 months of age, with a peak at around 2–3 months of age [14,15,16]. The half-lives of antibodies induced by vaccination of pregnant women with diphtheria, tetanus, and pertussis (DTP) vaccines are 28.7 days (tetanus toxoid antibodies) and 35.1 days (pertactin antibodies) [17], while those of RSV vaccines may be similar. Vaccination of pregnant women alone cannot cover the entire high-risk period of severe disease. Additionally, RSV vaccines may be capable of inducing antibody-dependent enhancement (ADE) of infection, as observed with dengue vaccines, if the antibody concentration in the blood decreases; therefore, this possibility needs to be examined.

We hypothesized that the efficacy and safety of the vaccine could be enhanced by inducing antibodies near the CCD of the G protein rather than just the F protein. KD-409 was designed to induce antibodies around the CX3C motif, anti-G162-171, as it lacks the CX3C motif and is therefore less likely to cause inflammation. Although not directly binding to the CX3C motif, this anti-G antibody is expected to act as a form of steric hindrance due to the bulkiness of the antibody itself, inhibiting the CX3C-CX3CR1 interaction. At the same time, the ability of KD-409 to induce anti-G antibodies was lower than that of anti-F antibodies, suggesting that other factors may contribute to its efficacy and safety. For example, KD-409 forms a particle structure, which is closer to post-F-like structure than pre-F-like structure. The immune response triggered by these characteristics may be one of the factors influencing efficacy and safety. Our previous report mentioned the possibility that the IgG2a/IgG1 ratio differed from that of pre-F owing to variations in particle size and shape, based on the results from mouse experiments. We are currently elucidating the mechanism, which will be reported in the future. In a previous report, we demonstrated that KD-409 outperformed pre-F in protecting against infection in a pregnant mouse model [18]. Although our study demonstrated efficacy in a mouse pregnancy model, we were unable to report safety or efficacy data in animals other than mice.

Therefore, in the present study, we evaluated the safety and efficacy of KD-409 in multiple rodent species. As a novel vaccine encompassing all age groups, i.e., from newborns to children, KD-409 may represent a next-generation vaccine with superior safety and efficacy compared to RSV F-based vaccines.

## 2. Materials and Methods

### 2.1. Antigens, Viruses, Cells, and Animals

Recombinant protein (pre-F and KD-409) and inactivated RSV (FI-RSV): Plasmid DNA was introduced into Expi293F cells (A14527, Thermo Fisher Scientific K.K., Tokyo, Japan) or CHO DG44 cells (Meiji Seika Pharma, Tokyo, Japan) and cultured with agitation to transiently express recombinant proteins. F protein sequences were derived from those of RSV A strains. Pre-F contains DS-Cav1 mutations, including S155C, S290C, S190F, and V207L. KD-409 has a portion of the FP domain of the F protein replaced by DFHFEVFNFV, which is derived from the G protein sequence. Site IV glycosylation of KD-409 was introduced through mutations K419N, K421T, T434N, and S436T in the F protein sequence. KD-409 has 6 × His-FLAG tags (obtained from Expi293F) or no tag (obtained from CHO DG44), while pre-F has a trimerization tag, Foldon, and 6 × His-Strep tags at the C-terminus. The FI-RSV was prepared based on the Lot 100 formulation manufactured by Pfizer, Inc. (New York, NY, USA) in the mid-1960s, as previously described [2,19]

Viruses: RSV A2 (VR-1540; ATCC, Manassas, VA, USA) was prepared as reported previously. HEp-2 cells (CCL-23; ATCC, Manassas, VA, USA) were seeded into flasks at 1 × 10^5^ cells/mL and pre-cultured at 37 °C with 5% CO_2_. The cells were cultured with RSV A2 in Minimum Essential Medium (MEM; Thermo Fisher Scientific K.K.) supplemented with 2% fetal bovine serum (FBS; Global Life Sciences Technologies Japan K.K., Tokyo, Japan) at 1–4.5 × 10^5^ cells/mL for 3 days at 37 °C and 5% CO_2_. After centrifugation (390× *g*, 5 min, 4 °C), the supernatant was collected, and flasks were washed with Dulbecco’s Phosphate-Buffered Saline (D-PBS). The cells were detached using 0.25% Trypsin/EDTA (Thermo Fisher Scientific K.K.) and collected in MEM supplemented with 2% FBS. After centrifugation (390× *g*, 5 min, 4 °C), the supernatant was removed, and the cell pellet was suspended in 4 mL of 25% sucrose/PBS. The cell suspension was frozen in liquid nitrogen, thawed, pipetted 50–60 times, and centrifuged (390× *g*, 5 min, 4 °C), after which the supernatant was collected.

Cells: FI-RSV and RSV A2 were obtained from Hep-2 (ATCC); pre-F was obtained from Expi293 (Thermo Fisher Scientific K.K.); and KD-409 was obtained from Expi293 or CHO DG44 (Meiji Seika Pharma).

### 2.2. Immunization

Mice (active): Six- to seven-week-old female BALB/c mice weighing 15 to 20 g (Japan SLC, Inc., Shizuoka, Japan) were administered two intramuscular doses of various antigens at 3-week intervals. Three weeks later, blood was collected under isoflurane anesthesia and centrifuged (3000× *g*, 10 min, 25 °C) to obtain serum.

Mice (passive): Pooled serum was prepared by mixing equal volumes of serum from 16 mice immunized twice at 3-week intervals with Adju-phos-administered Pre-F or KD-409 at 5 µg/dose. The sera of other actively immunized mice were diluted with phosphate-buffered saline and administered intraperitoneally.

Guinea pigs (pregnant): Pregnant Hartley guinea pigs (Japan SLC) were administered two intramuscular injections of antigens at 3-week intervals, and their blood, together with that of the pups, was collected 0, 15, and 30 days after delivery. The collected blood specimens were centrifuged (3000× *g*, 10 min, 25 °C) to obtain serum. Pooled serum was prepared by mixing equal volumes of serum from 5–9 guinea pigs.

Cotton rats (active): Female Hsd cotton rats (ENVIGO, Indianapolis, IN, USA/Japan SLC) aged 11–12 weeks were administered two intramuscular injections of antigens at 3-week intervals. The collected blood specimens were centrifuged (3000× *g*, 10 min, 25 °C) to obtain serum.

Note: Group sizes are indicated in the captions for each figure. Each animal experiment was thoroughly validated through multiple repetitions after careful examination of the conditions. Animals were randomly assigned. The laboratory technician and evaluator were different individuals, and the treatment group composition was blinded.

### 2.3. Enzyme-Linked Immunosorbent Assay (ELISA) and Cell-ELISA

Anti-F antibody titer: Serum was applied to 96-well Pierce nickel-coated plates (Thermo Fisher Scientific K.K., Tokyo, Japan), immobilized with pre-F, and blocked with 1% bovine serum albumin. Detection was performed using horseradish peroxidase-conjugated anti-mouse IgG (Global Life Sciences Technologies Japan K.K., Tokyo, Japan), anti-guinea pig IgG, anti-cotton rat IgG, and 3,3′,5,5′-tetramethylbenzidine liquid substrate (Sigma-Aldrich Co., St. Louis, MO, USA). The results were measured using SPECTRAMAX 190 (Thermo Fisher Scientific K.K.).

Neutralizing antibody titers: In brief, 2 × 10^5^ cells/mL HEp-2 cells (CCL-23; ATCC) were pre-cultured in 96-well plates. Serum and RSV diluent (4.4 × 10^2^–1.2 × 10^3^ pfu/well) containing rabbit serum complement (Cedarlane, Burlington, ON, Canada) were mixed in equal volumes and incubated at 37 °C and 5% CO_2_ for 1 h. After removing the culture supernatant from the plate, the serum-RSV reaction solution was added and incubated at 37 °C and 5% CO_2_ for 2 days. The infected cells were fixed in methanol, air-dried, and detected with an anti-F antibody, anti-mouse IgG-Alexa488 conjugated antibody (Abcam, Cambridge, UK), and Hoechst 33,342 solution (Dojindo Laboratories Co., Ltd., Kumamoto, Japan). The infection rate was analyzed using an image analyzer (Image Xpress Micro XLS; Molecular Devices, LLC., San Jose, CA, USA). The analysis method was as follows: the relationship between the serum dilution factor and the infection inhibition rate was plotted, and the infection rate of the wells to which only RSV was applied was considered to be 100%. The neutralizing antibody titer (IC_50_) was calculated by curve fitting in GraphPad Prism 9.5.1 (GraphPad Software Inc., San Diego, CA, USA) or set by the dilution factor that exceeded a 50% inhibition rate.

### 2.4. Virus Challenge and Copy Number Analysis

RSV A2 (1 × 10^5^–10^6^ PFU; Vero cell titration, within this range depending on the lot of the virus used) was inoculated intranasally (10–20 µL or 100 µL; mouse-active/passive or cotton rat) under 2–3% isoflurane anesthesia. Three days after viral challenge, whole lungs were collected in Lysing Matrix D tubes (MP-Bio Japan K. K., Tokyo, Japan), followed by the addition of TRIzol (Thermo Fisher Scientific K.K.). The tubes were agitated with FastPrep-24 (MP-Bio Japan K.K.), and the lung tissue was crushed. cDNA was synthesized from the extracted RNA using a High-Capacity cDNA Reverse Transcription Kit (Thermo Fisher Scientific). Quantitative polymerase chain reaction (PCR) was performed to detect the number of viral copies using the sense primer CARCAAAGTTAYTCTATCATGTC, antisense primer GATCCTGCATTRTCACARTACCA, minor groove binder (MGB) probe TGTAGTACAATTRCCACT, and standard DNA; TGTCCAACAATGTTCAAATAGTTAGACAGCAAAGTTACTCTATCATGTCCATAATAAAAGAGGAAGTCTTAGCATATGTAGTACAATTACCACTATATGGTGTTATAGATACACCCTGTTGGAAACTACACACATCCCCTCTATGTACAACCAACACAAAAGAAGGGTCCAACATCTGTTTAACAAGAACTGACAGAGGATGGTACTGTGACAATGCAGGATCAGTATCTTTCTTCCCACAAGCTGAAACATGTA. Real-time PCR cycling conditions were as follows: 50 °C for 2 min, initial denaturation at 95 °C for 10 min, followed by 50 cycles at 95 °C for 30 s and 60 °C for 1 min. A calibration curve was prepared from the amplification curve of the standard, and the virus copy numbers of the samples were calculated using QuantStudio 7 (Applied Biosystems, Waltham, MA, USA). Samples below the detection limit were analyzed at the lower limit of 100 copies.

### 2.5. Inflammation Assessment

Lung tissue section: BALB/c mice were immunized twice with antigen protein containing 0.5, 5, 50, or 500 ng/dose with Adju-Phos and challenged with RSV A2. Whole lungs were harvested 3 days after the challenge, and the left lung was fixed in 10% formalin. Paraffin-embedded tissues were sectioned at a thickness of 2.5 µm and stained with hematoxylin and eosin for histological evaluations. Four mice were assigned to each dose (n = 4), and three tissue sections were prepared for each mouse. The results of the group exhibiting the highest inflammation level were used. Pulmonary inflammatory symptoms (perivascular interstitial edema, peribronchial and perivascular cellular infiltration, alveolar wall thickening, and bronchial wall mucus) were evaluated by an experienced specialist using an Eclipse 80i microscope (NIKON CORPORATION, Tokyo, Japan).

Inflammatory cells: BALB/c mice were immunized twice with an antigen protein containing 5 ng/dose with Adju-Phos were challenged with RSV A2. The bronchoalveolar lavage fluid (BALF) was collected 3 days after the challenge, and cells were applied to glass slides using Cytospin 4 (PHC Corporation, Tokyo, Japan). The slides were stained with Diff-Quik (16920; SYSMEX CORPORATION, Kobe, Japan). The numbers of multinucleated cells, eosinophils, and neutrophils were counted by a trained specialist using ImageXpress pico (Molecular Devices, LLC., San Jose, CA, USA).

### 2.6. Statistical Analysis

Anti-RSV F antibodies, neutralizing antibody titers, and viral loads are presented as geometric means and 95% confidence intervals (CIs). The ratios of polymorphonuclear (PMN) leukocytes, eosinophils, and neutrophils are presented as arithmetic means and 95% CIs. Statistical analysis was performed using GraphPad Prism version 9.5.1 (GraphPad Software Inc.). Neutralizing antibody titers and virus copy numbers were analyzed using the Kruskal–Wallis test and Dunn’s multiple comparison test. To evaluate inflammation, the Brown-Forsythe test, Welch’s ANOVA, and Dunnett’s T3 multiple comparison test were used. Nonparametric tests were adopted that does not assume any specific distribution of the data, based on the size of the population.

## 3. Results

### 3.1. Antibody Transfer in a Guinea Pig Pregnancy Model

KD-409 was designed to be a safe and effective vaccine for all children, including newborns and infants. To achieve this, we verified the potential challenges for each target group using animal models. For newborns, the prevention of severe disease relies on maternal antibodies transferred through maternal vaccination. This is because the risk of severe disease is high within the first few months of life, and conventional active immunization may not allow for sufficient time for the immune system to mature. Additionally, infants have immature immune systems, and active immunization may not induce sufficient neutralizing antibody titers. If sufficient immune induction does not occur, the possibility of ADE cannot be ruled out, necessitating verification. While future human verification is required, we attempted verification in animal models as close to humans as possible during the preclinical stage. For the maternal antibody model, we selected guinea pigs, which share the same placental antibody transfer mechanism as humans. However, since guinea pigs have limited established use as a human RSV infection model, we focused only on evaluating maternal antibodies and did not assess infection protective efficacy. The guinea pig is an appropriate model for mimicking human antibody transfer [20]. Nonclinical studies on RSV vaccines have been conducted in guinea pigs, and antibody transfer has been confirmed for F protein-only antigens [21].

**Figure 1:**
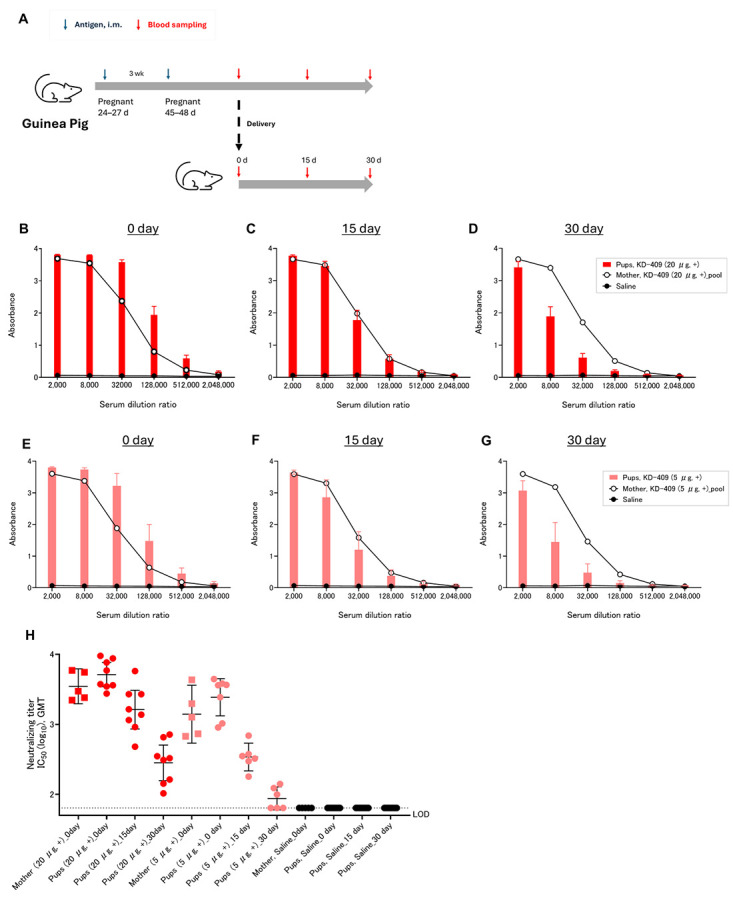
Neutralizing anti-F antibodies transfer from mother to pup in a guinea pig pregnancy model. (**A**) Experimental flow diagram of the guinea pig pregnancy model. Mother and pup sera were collected 3 weeks after two intramuscular immunizations with KD-409 at 3-week intervals. (**B**–**D**) Anti-RSV F antibody titer in the pup serum and mothers’ pooled serum (n = 5–9; days 0, 15, and 30) from animals immunized with a 20 µg/dose. (**E**–**G**) Anti-RSV F antibody titer in the pup serum and mothers’ pooled serum (n = 5–9; days 0, 15, and 30) from animals immunized with a 5 µg/dose. (**H**) Neutralizing antibody titer in the pup and mothers’ serum (n = 5–9; days 0, 15, and 30).

We employed a guinea pig pregnancy model system to confirm the antibody transfer of KD-409, which is a chimeric FG protein containing a part of the G protein (Figure 1A). Anti-F antibodies were detected in the serum of guinea pigs that had been immunized twice, as well as in the serum of their pups born to respective mothers on day 0 (day of birth = day 0) (Figure 1B,E). On day 0, the titers of both anti-F and neutralizing antibodies were higher in the pups than in the mother, indicating that the antibody concentration could be attributed to the transfer of antibodies from the mother to the pups (Figure 1B,E,H). The dose-dependent induction of anti-F and neutralizing antibodies was confirmed by comparing immunizations with 20 and 5 µg (Figure 1B–H). These results indicated the occurrence of antibody transfer in the guinea pig model.

### 3.2. Evaluation of Infection Exacerbation Following Passive Immunization in Mice

In vaccine safety evaluations, it is crucial to examine the potential for adverse events due to VED and ADE. Although the safety of RSV vaccines has been assessed using ex vivo infection enhancement validation [22], few studies have evaluated infection enhancement using animal models.

**Figure 2:**
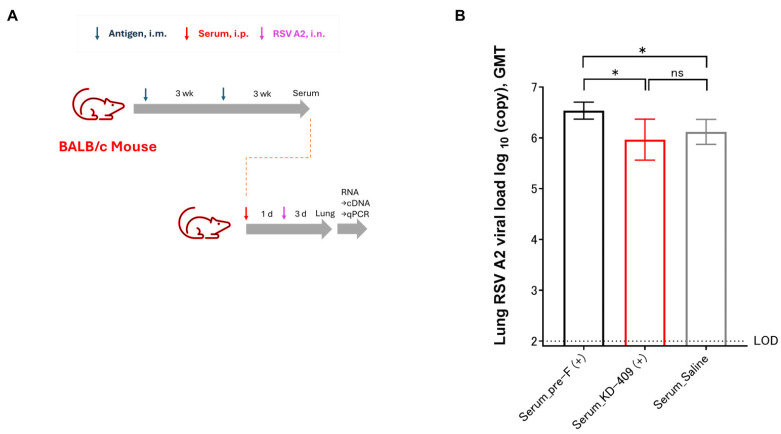
KD-409 with Adju-Phos does not exacerbate infection in passively immunized mice. (**A**) Experimental flow diagram of mouse passive immunization (immunized serum-challenged model). The serum was obtained after two intramuscular immunizations with KD-409 at 3-week intervals. Mice were challenged with RSV 1 day after intraperitoneal administration (i.p.), and lung tissues were harvested 3 days later. (**B**) Comparison of the virus copy numbers of passively immunized mice (immunized serum administration). Statistical analysis was performed by one-way-ANOVA and Dunn’s multiple comparison test; n = 13; * *p* < 0.05, ns: *p* > 0.05, not significant. LOD: Limit of detection.

We evaluated the safety of a passive mouse immunization model (Figure 2A). The occurrence of VED or ADE is thought to be owing to a lack of sufficient neutralizing antibodies. Considering the immune serum-administered infection model, we obtained serum samples after two immunizations and determined the serum dilution factor at which the neutralizing capacity was reduced. The anti-F antibody concentrations in serum after two immunizations with Pre-F or KD-409 were approximately equivalent at 385.2 ± 44.2 and 362.2 ± 43.85 μg/mL (mean ± S.D.), respectively (values measured from pooled immune sera obtained from n = 16 subjects). In addition, the KD-409 immune serum contained anti-G antibodies at a concentration of 3.1 ± 0.4 µg/mL (mean ± S.D.). We administered 0.4 mL of Pre-F or KD-409 immune serum diluted at 10^−1^, 10^−2^, 10^−3^, 10^−4^, 10^−5^, 10^−6^, 10^−7^, 10^−8^, 10^−9^, and 10^−10^ into the peritoneal cavity and preliminarily confirmed that infection-preventive activity was lost at dilutions exceeding 10^−7^. Furthermore, when 0.4 mL of pre-F and KD-409 immune serum diluted 10^−7^, 10^−8^, 10^−9^, and 10^−10^ was administered intraperitoneally to compare their preliminary infection-preventing abilities, interestingly, a difference was observed at the 10^−8^ dilution condition. When pre-F and KD-409 immune sera were diluted 10^8^-fold and administered at 0.4 mL, the anti-F antibody titers were 1.54 pg or 1.45 pg, respectively, showing little difference. Upon re-verifying under these conditions, that is, mice treated with serum diluted to factor 10^8^ were challenged with RSV, and the number of viral copies in the lung tissue was compared. The results showed that pre-F significantly increased (*p* = 0.0378) the number of viral copies compared to saline, whereas KD-409 showed results comparable to those of saline (Figure 2B). Overall, this result suggested that pre-F, but not KD-409, exacerbated infection in the mouse passive immunization model.

### 3.3. Evaluation of Symptom Exacerbation Following Active Immunization in Mice

**Figure 3:**
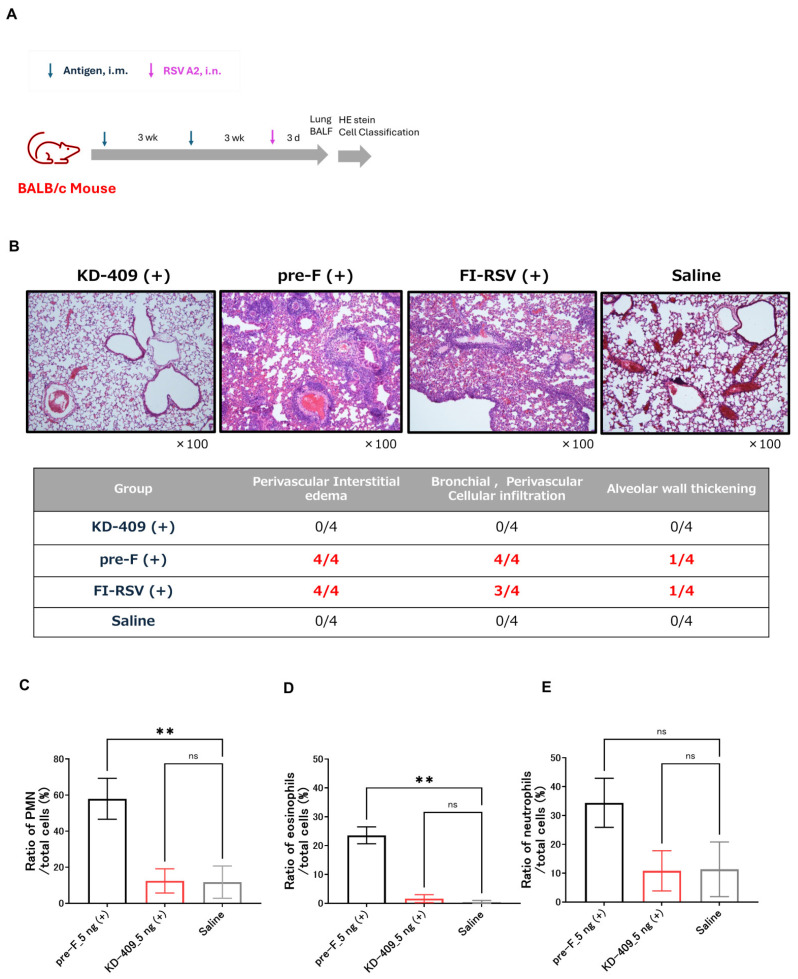
KD-409 with Adju-Phos does not exacerbate symptoms in actively immunized mice. (**A**) Experimental flow diagram of mouse active immunization. After two immunizations with KD-409 at 3-week intervals, mice were challenged with RSV, and the lungs were harvested 3 days later. (**B**) Representative images of hematoxylin-eosin-stained tissue sections and evaluation of inflammation in lung tissue sections. Pathology image: KD-409 (5 ng), Pre-F (5 ng), and FI-RSV (500 ng). The proportion of mice showing the most symptoms of exacerbated inflammation (n = 4; 0.5, 5, 50, and 500 ng/dose). (**C**–**E**) Ratio of polymorphonuclear (PMN) leukocytes, eosinophils, and neutrophils in the bronchoalveolar lavage fluid (BALF) (n = 3). Statistical analysis was performed by one-way-ANOVA and Dunnett T3 multiple comparison test. ** *p* < 0.01, ns: *p* > 0.05, not significant.

The licensed vaccine for pregnant women consists of adjuvant-free pre-F protein antigen, and no safety concerns have been reported in clinical trials. In contrast, no licensed RSV vaccine has been approved for use in children. Several animal studies have shown that active immunization with alum-adjuvanted pre-F can cause pulmonary inflammation, which is a concern for the development of a pediatric vaccine. The safety concerns with the pediatric RSV vaccine are due to the fact that the target population for vaccination is Th2-dominant and the adjuvant induces a Th2-dominant immune response. BALB/c mice are Th2 dominant compared to C57BL/6 mice and Adju-Phos adjuvant has been reported to induce Th2 dominant immunity. RSV infection in the absence of adequate immunity or weakened blood antibody titers may result in the exacerbation of infection and symptoms. We performed a safety evaluation using an active immunization model in which BALB/c mice were immunized with a low dose of RSV (Figure 3A). We analyzed the number of viral copies and found that FI-RSV immunization resulted in infection exacerbation (Figure S1). Given that infection was exacerbated upon administering a 50 ng/dose of FI-RSV containing Adju-Phos, the antigen protein dose was set at 0.5, 5, 50, or 500 ng. To evaluate whether exacerbation of infection and symptoms occurred at the same dose, and whether the dose at which symptoms exacerbated differed depending on the antigen, we decided to allow a range of doses to be administered. The results showed the dose at which the most significant inflammation was observed within this dose range. Lung tissue inflammation was observed at 500 ng/dose for FI-RSV with Adju-Phos and 5 ng/dose for pre-F with Adju-Phos, but no lung tissue inflammation was observed at any dose for KD-409 with Adju-Phos (Figure 3B). Adju-phos-containing pre-F showed lung inflammation; therefore, as a follow-up experiment, we evaluated inflammation at the cellular level when mice were infected after two immunizations at 5 ng/dose. Additionally, the number of PMN leukocytes, eosinophils, and neutrophils in the BALF was significantly higher in the pre-F with Adju-Phos group than the saline groups; however, those of KD-409 with Adju-Phos were similar to those in the saline group (Figure 3C–E). These results suggested that pre-F with Adju-Phos is more likely to exacerbate symptoms in the absence of adequate immunity, whereas KD-409 is less likely to exacerbate symptoms.

### 3.4. Efficacy Evaluation by Active Immunization of Cotton Rats

**Figure 4:**
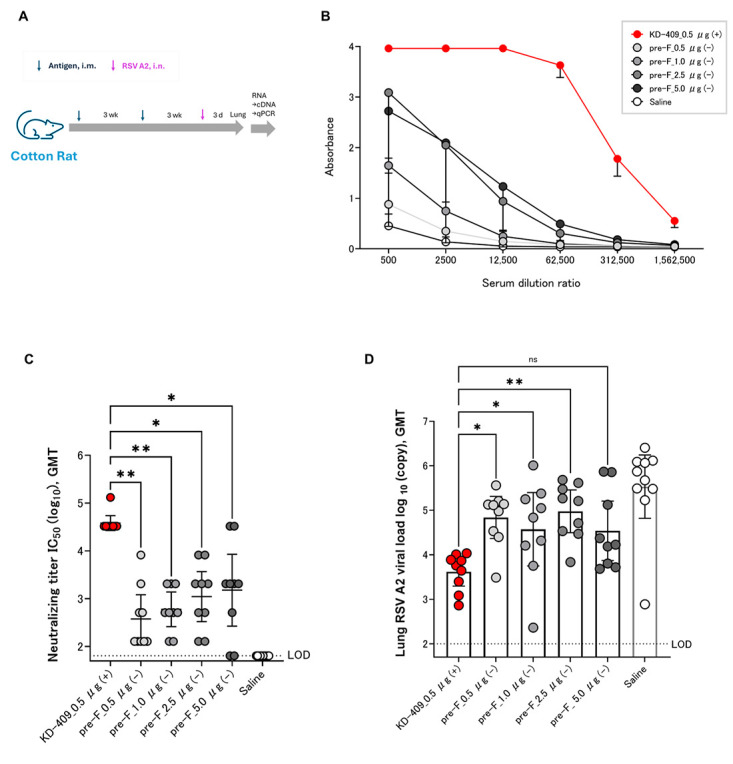
Low-dose KD-409 with Adju-Phos has greater efficacy than adjuvant-free high-dose pre-F in the cotton rat model. (**A**) Experimental flow diagram of the cotton rat model; following two intramuscular injections of KD-409 at 3 week-intervals, the lungs were harvested 3 days after RSV challenge (n = 9–10). Comparison of Adju-Phos-containing KD-409 and adjuvant-free pre-F; (**B**) anti-F antibody titer (n = 9–10); (**C**) neutralizing antibody titer; and (**D**) protective efficacy against infection. Statistical analysis was performed by one-way-ANOVA and Dunn’s multiple comparison test; n = 9–10; ** *p* < 0.01, * *p* < 0.05, ns: *p* > 0.05, not significant.

The addition of alum-based adjuvants to pre-F suggested the possibility of exacerbating infection in passive immunity and exacerbating symptoms in active immunity (Figure 2 and Figure 3). In particular, the active immunity of vaccines that combine alum adjuvant and pre-F for children cannot completely eliminate concerns about VED, such as lung inflammation. The vaccine formulation approved for pregnant women consists of pre-F protein antigen without Th2-inducing adjuvant. This adjuvant-free pre-F formulation may represent a safe RSV vaccine for children. We previously evaluated protection against infection using a mouse model [18]. Herein, we evaluated efficacy in a cotton rat model, which allows for a higher degree of extrapolation to humans (Figure 4A). Upon incorporating the Adju-Phos adjuvant in the formulation, we found that pre-F and KD-409 were comparable in terms of efficacy (Figure S2); however, pre-F with alum-based adjuvant is unlikely to be used as a pediatric formulation due to safety concerns. KD-409 with Adju-Phos had a low risk of infection and symptom exacerbation (Figure 2 and Figure 3), suggesting that some of the safety profile of KD-409 has been confirmed in mouse models. We compared the efficacy of adjuvant-free Pre-F and Adju-Phos-added KD-409 as potential future pediatric formulations.

To confirm dose-dependent immunogenicity and efficacy, we measured the anti-F and neutralizing antibody titers in blood and viral copy numbers in lung tissue after RSV challenge. Immunization with a 0.5–5.0 µg/dose of KD-409 induced dose-dependent anti-F antibody titers; the adjuvanted antigen amount of 1/10 of KD-409 had a higher anti-F antibody induction ability than the adjuvant-free pre-F (Figure 4B). In terms of neutralizing antibody titers, KD-409 with adjuvant significantly outperformed pre-F without adjuvant, despite a 1/10 antigen dose (Figure 4C). Assessing protection against infection in the cotton rat model, KD-409 with an adjuvant significantly outperformed pre-F without an adjuvant, which was five times the amount of antigen. Adjuvanted KD-409 reduced viral copy numbers (GMT) compared to the unadjuvanted pre-F tenfold antigen dose (Figure 4D). These results suggest that KD-409 has superior efficacy to adjuvant-free pre-F in the cotton rat model.

## 4. Discussion

**Figure 5:**
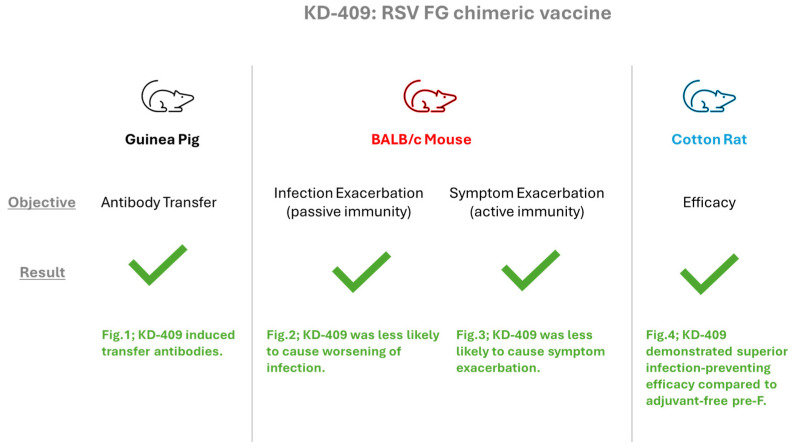
Summary of KD-409 evaluation in animal models. Antibody transfer was evaluated in guinea pigs, and passive immunization-induced infection exacerbation and active immunization-induced symptom exacerbation were evaluated in BALB/c mice and cotton rats.

In this study, we evaluated the safety and efficacy of KD-409, a chimeric RSV FG protein that incorporates a highly conserved region of the RSV G protein, in various rodent species (Figure 5). In a passive immunization model in which pregnant females were inoculated, we found that antibody transfer occurs in guinea pig models and that KD-409 is less likely to exacerbate infection in a mouse passive model than conventional antigens such as FI-RSV and pre-F with Adju-Phos. In a low-dose mouse model of active immunization that mimics infant vaccination and fails to induce sufficient immunity, KD-409 with Adju-Phos was less likely to exacerbate inflammation than FI-RSV or pre-F with Adju-Phos. In a cotton rat model, which is a well-established method for evaluating the efficacy of human RSV vaccines, KD-409 was significantly more effective in eliciting protection against infection than unadjuvanted pre-F.

Currently, there is no RSV vaccine that covers from neonates to children and is both effective and safe. When pregnant women are vaccinated, transferable antibodies from the mother to the neonate are expected to decline over time. Regardless of vaccination, the neutralizing antibody titer at 3 months of age is less than 1/10 of that at 0 days of age [23], and 60% of hospitalizations due to RSV infection in children under 1 year of age occur within the first 3 months of life [24]. Moreover, vaccination of children may not induce sufficient immunity because of the characteristics of childhood immunity [25]. Additionally, vaccination of pregnant women alone is not sufficient to prevent severe illness and hospitalization in newborns, infants, and children. RSV vaccines need to be developed for both newborns with decreasing maternal antibodies and children with immature immune systems. VED first occurred approximately half a century ago [2], and vaccine development for RSV in children has progressed with caution, with no licensed vaccines as yet. Therefore, vaccine efficacy and safety concerns exist and need to be addressed for both maternal and pediatric vaccines.

We previously demonstrated the immunogenicity and efficacy of KD-409 in mouse models of active and passive immunization [18]. In the current study, we focused on the safety of KD-409 for maternal and pediatric immunization and compared its efficacy to that of the approved formulation, pre-F, without an adjuvant for maternal immunization. In the case of tetanus and pertussis vaccines administered to pregnant women, the half-live of transferable antibodies is approximately 30 days for both [17]. The half-life of the maternal RSV antibody is 36–42 days [26], and the neutralizing titer of the transfer antibody induced by RSVPreF3 maternal immunization decreases by half within 43 days [23]. In the guinea pig models, with the alum-adjuvanted RSV recombinant F nanoparticle vaccine, anti-F antibody and neutralizing antibody titers were halved within 30 days of birth [21], consistent with our findings (Figure 1H). The half-life of transplacental antibodies induced by KD-409 is expected to be similar to that of conventional maternal immunization. When antibodies are transferred from mother to newborns, the concentration of antibodies increases, a process known as antibody concentration [27]. In our experiment, the anti-F antibody titer (GMT) of the pups was higher than that of the mother on day 0. This result suggests that the concentration of transitional antibodies induced by KD-409 is similar to that induced by conventional maternal immunization.

Cases of severe RSV infection in infants requiring hospitalization peak at approximately 2–3 months of age, and the risk of severe RSV infection is also high in those whose transient antibodies have lost their effectiveness and in children under 5 years of age [1,15,16,28,29]; hence, an age-appropriate vaccine is urgently needed. Children have immature immune systems, and vaccination may fail to induce sufficient antibody titers [30]. Ex vivo and in vitro studies have suggested that inadequate immunity may result in low neutralizing antibody titers, increasing the likelihood of enhanced ADE infection [31]. In an active immunization model of cotton rats, low-dose immunization with the F antigen alone demonstrated a trend toward the exacerbation of symptoms after vaccination with post-F or pre-F with alum [32]. In addition, immunization with pre-F-alum exacerbated symptoms in a BALB/c mouse model [9]. A new and simplified in vivo system is required to evaluate infection-induced adverse reactions and symptom exacerbation after vaccination. In this study, we demonstrated that KD-409 with Adju-Phos is unlikely to cause infection and symptom exacerbation after vaccination using a mouse passive immunization model and a low-dose mouse active immunization model (Figure 2 and Figure 3). Lung sections from infants who died in the 1967 FI-RSV clinical trial revealed respiratory eosinophils and CCL5 as markers of enhanced RSV disease [33]. In this study, we focused on eosinophils and evaluated them in a mouse model, demonstrating that pre-F-Adju-Phos increased eosinophil counts, whereas KD-409-Adju-Phos did not.

We also demonstrated that KD-409 was less likely to exacerbate infections and symptoms (Figure 2 and Figure 3), and discuss the mechanism of exacerbation suppression. CX3C in RSV G suppresses the function of normal Th1 cells by interacting with CX3CR1 in the host cell, whereas anti-G antibody promotes Th1 cell function by inhibiting this interaction [34]. In a cell migration assay, 3D3 antibodies functionally inhibited the CX3C-CX3CR1 interaction [35]. As reported previously, KD-409 inoculation results in Th1 dominance; however, the induced anti-G antibody titer remains relatively low [18], making it difficult to verify in the cell migration system. Whether low anti-G antibody levels are a significant factor in the inhibition of exacerbation remains debatable. We believe that the various immune responses to KD-409 need to be examined; hence, we are actively acquiring additional data.

The occurrence of infection and symptom exacerbation may depend on the antibody concentration in the blood; however, it is difficult to measure all antibody concentrations comprehensively, which is a limitation of this experimental system. The epitope population of anti-F antibodies induced by G-sequence insertion is potentially altered. However, in low-dose immunization, the antibody induction capacity was low, suggesting that other factors may have a greater impact than antibody quality. CD4^+^ T lymphocytes have also been associated with VED [8]. Although we focused on the analysis of inflammatory cells in BALF, in the future, we would like to conduct more comprehensive and detailed analyses using flow cytometry and other equipment to elucidate the full mechanism of VED. Moreover, whether the infection exacerbation was a direct cause of the symptom exacerbation could not be determined. We believe that detailed analysis of the immune response is necessary to determine whether worsening infection leads to increased symptoms. ABRYSVO, approved for vaccination of pregnant women, is an adjuvant-free pre-F. This approved vaccine contains bivalent pre-F antigens against RSV A and B strains mutated at 847aa to enhance conformational stability and immunogenicity [36]. This antigen differs from previously reported DS-Cav1 mutants, including S155C, S290C, S190F, and V207L [37]. It is important to note that the pre-F used in our experiments is a monovalent DS-Cav1 mutant vaccine derived from the RSV A strain; therefore, our results are not strictly comparable to those of the licensed vaccine (Figure 4). In September 2024, Moderna announced that it had discontinued its clinical trial of an RSV mRNA vaccine for infants aged 5–24 months. The FDA revealed that children who received the vaccine in clinical trials had a higher incidence of severe RSV infections compared to children in the placebo group. Approximately 12.5% of vaccinated children developed severe or very severe RSV infection, compared to only 5% in the placebo group. Furthermore, among those who developed symptomatic RSV infection, 26.3% of vaccinated participants developed severe disease, compared to 8.3% in the placebo group. These exacerbations were caused by the new modality, mRNA-LNP, though the reason remains unclear. This is expected to impact other RSV vaccines and current and future pediatric development programs, and we will continue to strive to identify the cause [38]. RSV A and B strains have been circulating in the community. Here, we have presented data on the RSV A strain, and will proceed with acquiring data on the B strain going forward. The experimental conditions for the two doses in this study and the condition of mothers with no history of infection are not completely identical to those for actual human vaccination. The first dose is assumed to replace immunity acquired through infection, but it is important to note that accurate evaluation using a previously infected model would be preferable. Cases of severe illness in children are associated with primary infection. We validated this using naïve animals simulating children without any prior infections under 2 years of age but have not validated it using a pre-infected model simulating children with a history of infection up to approximately 5 years of age. Given that the Th2-primed immune system is predominant in neonates and children [39], and that BALB/c mice are Th2-predominant compared to C57BL/B6 mice [40], we used this animal model to mimic pediatric vaccination in this experiment. However, the biological indicators, except for Th2 dominance, differ from those of human newborns and children. We note that while RSV infects rodents, the clinical symptoms and pathology in humans and cattle are not necessarily the same. Moreover, although the cotton rat model is an established RSV vaccine evaluation system, from the perspective of human extrapolation, a monkey model is more desirable for evaluating safety and efficacy [41]. To verify the high safety and efficacy of the vaccine, studies using a monkey model will be necessary in the future, considering extrapolation to humans.

In conclusion, we evaluated the next-generation RSV vaccine KD-409 in rodent models and demonstrated that its safety and efficacy were superior to those of the pre-F antigen. Accordingly, KD-409 may be suitable for both maternal and pediatric vaccination. KD-409 is a promising next-generation RSV vaccine candidate for neonates and children.

## Data Availability

The datasets presented in this article are not readily available because the data are part of an ongoing study. Requests to access the datasets should be directed to the corresponding author.

## Supplementary Materials

The following supporting information can be downloaded at: https://www.mdpi.com/article/10.3390/vaccines13111170/s1, Figure S1: Evaluation of infection exacerbation after FI-RSV immunization.; Figure S2: Efficacy comparison with adjuvant-added pre-F.

## Abbreviations

The following abbreviations are used in this manuscript:

RSV	Respiratory syncytial virus
ALRI	Acute lower respiratory infection
FI-RSV	Formalin-inactivated respiratory syncytial virus
VED	Vaccine-enhanced disease
FDA	Food and Drug Administration
CCD	Conserved central domain
BBG2Na	Fusion protein of RSV G protein and streptococcal G protein albumin-binding domain
DTP	Diphtheria, tetanus, and pertussis
ADE	Antibody-dependent enhancement
CX3C	Specific motif sequence in G protein
CX3CR1	CX3C chemokine receptor 1
PMN	Polymorphonuclear leukocytes
BALF	Bronchoalveolar lavage fluid
ELISA	Enzyme-linked immunosorbent assay
HEp-2	Human epithelial type 2 cells
PFU	Plaque-forming units
MGB	Minor groove binder
PCR	Polymerase chain reaction
ANOVA	Analysis of variance
