# Supplementary material for: Evaluation of the Safety and Efficacy of the Respiratory Syncytial Virus FG Chimeric Vaccine KD-409 in Rodent Models for Maternal and Pediatric Vaccination
Source: Vaccines (Basel). 2025 Nov 18;13(11):1170. doi: 10.3390/vaccines13111170 (PMC12656842; doi:10.3390/vaccines13111170)
Supplement: Supplementary file 1 [file vaccines-13-01170-s001.zip › vaccines-3928762-supplementary.pdf]

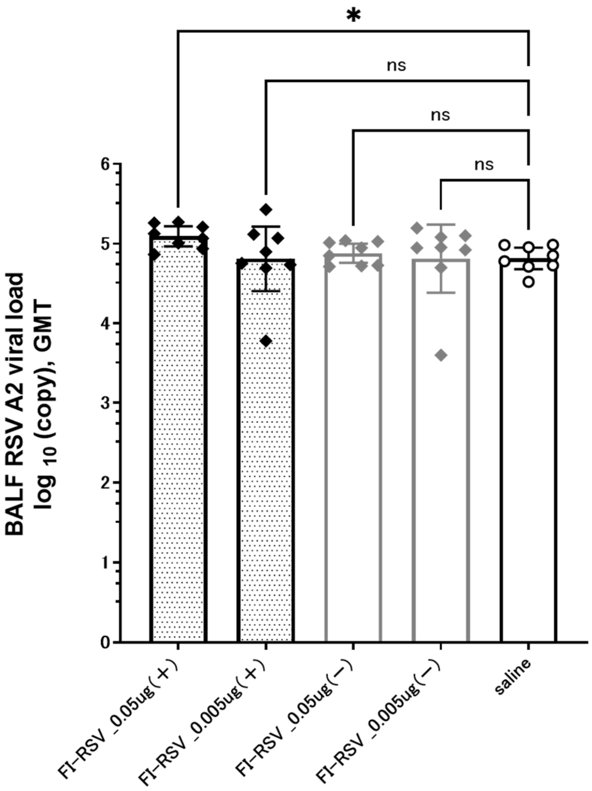

Figure S1. Evaluation of infection exacerbation after FI-RSV immunization. The immune conditions that exacerbated infection were investigated with and without adjuvants. After immunization with FI-RSV, the bronchoalveolar lavage fluid (BALF) was analyzed to determine the number of viral copies. After two immunizations with FI-RSV (Adju-Phos (+) or (−)) at 0.05 μg and 0.005 μg/doses, the dose that causes a decrease in neutralizing antibody titers and the number of viral copies in lung tissue were evaluated 3 days after RSV infection. Statistical analysis was performed using a one-way ANOVA followed by Dunn's multiple comparison test. $n = 9$; *$p < 0.05$, ns: $p > 0.05$, not significant.

.

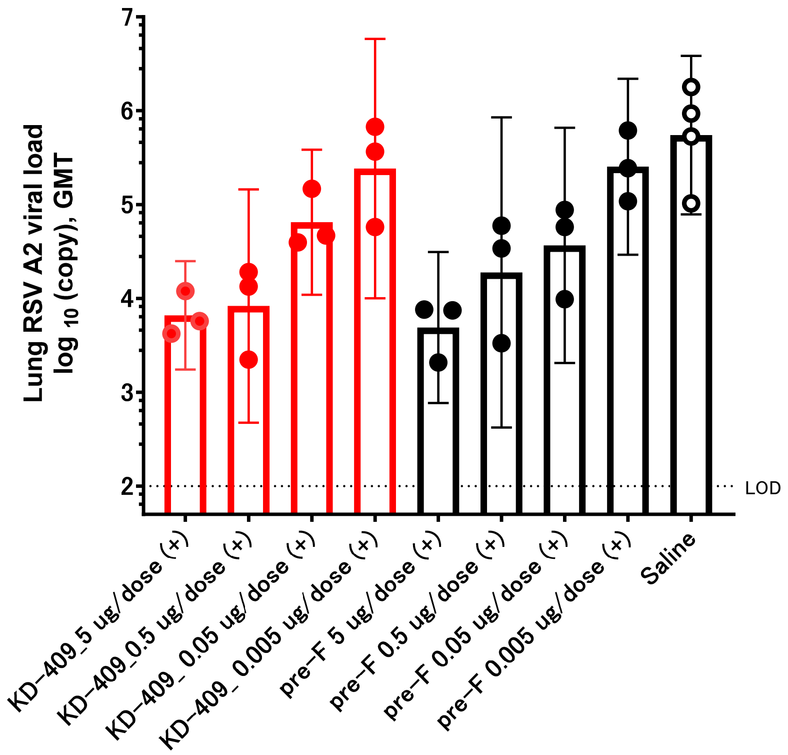

Figure S2. Efficacy comparison with adjuvant-added pre-F. The dose-dependent effects of KD-409 and pre-F in the presence of adjuvants were investigated. Adju-Phos-added KD-409 or pre-F was administered twice, followed by challenge with RSV A2, and the viral copy numbers in lung tissue were compared. The doses were 0.005, 0.05, 0.5, and 5 μg, with n = 3 in each group. The administration interval was 3 weeks and the viral copy number in the lung tissue was evaluated 3 weeks after the second administration.
